# Cortical brain volume abnormalities associated with few or multiple neuropsychiatric symptoms in Alzheimer’s disease

**DOI:** 10.1371/journal.pone.0177169

**Published:** 2017-05-08

**Authors:** Lyssandra dos Santos Tascone, Martha E. Payne, James MacFall, Dionísio Azevedo, Claudio Campi de Castro, David C. Steffens, Geraldo F. Busatto, Cássio M. C. Bottino

**Affiliations:** 1 Old Age Research Group–PROTER, Institute and Department of Psychiatry, University of Sao Paulo, Sao Paulo, Brazil; 2 CAPES Foundation, Ministry of Education of Brazil, Brasilia, DF, Brazil; 3 Office of Research Development, Duke University School of Medicine, Durham, North Carolina, United States; 4 Department of Radiology (Retired), Duke University School of Medicine, Durham, North Carolina, United States; 5 Department of Diagnostic Imaging, Heart Institute–InCor, Hospital das Clínicas at University of Sao Paulo, Sao Paulo, Brazil; 6 Department of Psychiatry, University of Connecticut Health Center, Psychiatry, Farmington, Connecticut, United States; 7 Laboratory of Psychiatric Neuroimaging, Department and Institute of Psychiatry, Faculty of Medicine, University of Sao Paulo, Sao Paulo, Brazil; Banner Alzheimer's Institute, UNITED STATES

## Abstract

New research on assessing neuropsychiatric manifestations of Alzheimer´s Disease (AD) involves grouping neuropsychiatric symptoms into syndromes. Yet this approach is limited by high inter-subject variability in neuropsychiatric symptoms and a relatively low degree of concordance across studies attempting to cluster neuropsychiatric symptoms into syndromes. An alternative strategy that involves dichotomizing AD subjects into those with few versus multiple neuropsychiatric symptoms is both consonant with real-world clinical practice and can contribute to understanding neurobiological underpinnings of neuropsychiatric symptoms in AD patients. The aim of this study was to address whether the number of neuropsychiatric symptoms (i.e., presence of few [≤2] versus multiple [≥3] symptoms) in AD would be associated with degree of significant gray matter (GM) volume loss. Of particular interest was volume loss in brain regions involved in memory, emotional processing and salience brain networks, including the prefrontal, lateral temporal and parietal cortices, anterior cingulate gyrus, temporo-limbic structures and insula. We recruited 19 AD patients and 13 healthy controls, which underwent an MRI and neuropsychiatric assessment. Regional brain volumes were determined using voxel-based morphometry and other advanced imaging processing methods. Our results indicated the presence of different patterns of GM atrophy in the two AD subgroups relative to healthy controls. AD patients with multiple neuropsychiatric manifestations showed more evident GM atrophy in the left superior temporal gyrus and insula as compared with healthy controls. In contrast, AD subjects with few neuropsychiatric symptoms displayed more GM atrophy in prefrontal regions, as well as in the dorsal anterior cingulate ad post-central gyri, as compared with healthy controls. Our findings suggest that the presence of multiple neuropsychiatric symptoms is more related to the degree of atrophy in specific brain networks rather than dependent on the global severity of widespread neurodegenerative brain changes.

## Introduction

Neuropsychiatric symptoms are highly prevalent when there is an established diagnosis of a dementing condition, such as Alzheimer´s Disease (AD) [1,2,3]. The identification and treatment of neuropsychiatric symptoms in dementia is essential because these symptoms are associated with decreased quality of life [4], worse prognosis, increased caregiver burden, greater impairment in activities of daily living [5], and increased risk of institutionalization [6]. The most commonly used scale to assess the presence and severity of neuropsychiatric symptoms in association with dementia is the Neuropsychiatric Inventory (NPI) [7].

Neuroimaging studies in patients with dementia may help to identify the neurobiological underpinnings of the emergence of NPI-assessed neuropsychiatric manifestations in AD, linking neuropsychiatric symptoms to the neurodegenerative process that characterize AD. This approach has been applied in a number of structural neuroimaging studies using magnetic resonance imaging (MRI), several of which have used voxel-based morphometry (VBM) methods that allow voxel wise inspections of foci of gray matter (GM) volume abnormalities across the entire brain, in an automated fashion [8,9,10,11,12,13]. Such VBM investigations of AD patients have reported significant associations between regional GM atrophy and the presence of each of the neuropsychiatric symptoms assessed by the NPI, including delusions [8,9,10,12], hallucinations [11], agitation [8,13], disinhibition [9], aberrant motor behaviour [13], depression [13] and apathy [8]. The associations between neuropsychiatric symptoms and brain volume abnormalities highlighted in these VBM studies have a variety of results, including: psychotic symptoms, agitation and disinhibition related to GM volume deficits in regions previously implicated in salience brain networks (namely the insular and anterior cingulate cortices) [14,15], as well as portions of the frontal, temporal, parietal and occipital cortices and the hippocampus [8,9,12,13]; and apathy and/or depression associated with GM atrophy in frontal and cingulated regions relevant to emotional processing [8,11,13].

Rather than assessing each neuropsychiatric manifestation in isolation, the grouping of neuropsychiatric symptoms in AD into syndromes is becoming widely used, with the aim of facilitating detection of prevalence rates of neuropsychiatric symptoms over time, associations with psychosocial determinants [16], and assessment of biological correlates [16,17]. However, inter-subject variability in regard to the presence of different neuropsychiatric symptoms is high [18,19] and there has been a relatively low degree of concordance across studies of the overall cluster composition from NPI symptoms into syndromes [17,19]. One alternative research strategy, not often explored, consists of dichotomizing AD subjects into those with few versus multiple neuropsychiatric symptoms [20]. Such a strategy is consonant with real-world clinical practice, since AD patients with neuropsychiatric symptoms often present several of those manifestations in combination [21], and it is challenging to model such clinical reality using statistical techniques that group symptoms into specific syndromes.

The present VBM study investigated the neural correlates of neuropsychiatric symptoms in dementia by dichotomizing a group of AD patients into those presenting few (two or fewer) symptoms versus those AD subjects presenting three or more types of neuropsychiatric manifestations as assessed with the NPI. Our exploratory hypothesis was that the presence of few versus multiple neuropsychiatric symptoms in AD would be associated with different degrees of significant GM volume loss in brain regions involved in memory, emotional processing and salience brain networks, including the prefrontal, lateral temporal and parietal cortices, anterior cingulate gyrus, temporo-limbic structures, and insula [8,9,22,23].

## Materials and methods

### Ethics statements

All procedures related to subjects´ selection, informed consents, collection of clinical and neuroimaging data and data analysis of this study were approved by the research ethics committee of the Hospital das Clínicas—School of Medicine, University of Sao Paulo, Brazil, under protocol numbers 408/01 and 0009/08. This local research ethics committee follows standard regulations and guidelines of the Ethics Committee of Research of the National Health Council, Ministry of Health, Federal Government of Brazil (CONEP). As the subjects invited to take part in the study could present cognitive deficits that impaired their full capacity to consent, both subjects and their relatives provided written informed consent. Regarding ethics requirements specifically data analysis, the preprocessing and analysis steps of anonymized neuroimaging data were approved by Duke University Health System (DUHS) Institutional Review Board (IRB) for Clinical Investigations under protocol number Pro00035156. The researcher who conducted those neuroimaging preprocessing and analysis steps (LST) was approved on the Biomedical Research course of the Collaborative Institutional Training Initiative (CITI) Program, with completion record number 7247606.

### Sample

The AD sample recruited for the present study was composed of 19 patients with mild dementia to moderate dementia: 9 from a community-based study conducted in Sao Paulo, Brazil [24,25], and 10 from an outpatients Old Age Psychiatry Dementia Clinic at the Hospital das Clínicas, School of Medicine, University of Sao Paulo (USP), Sao Paulo, Brazil. The recruitment of subjects took place between January 2003 and December 2004. All patients and their caregivers underwent a semi-structured interview with a psychiatrist or a neurologist at USP. The diagnostic evaluation of dementia included a complete medical history, physical and neurological examination, structural MRI scanning, laboratory workup for the differential diagnosis of dementia, the administration of the CAMDEX (Cambridge Mental Disorders of the Elderly Examination) [26, 27, 28] and its brief neuropsychological testing (CAMCOG—Cognitive Section of the CAMDEX), Mini Mental State Examination (MMSE) [29], Ischemic Hachinski Scale [30], and severity assessment as evaluated by the Clinical Dementia Rating Scale (CDR) [31, 32].Patients were included when they met DSM-IV [33] and NINCDS-ADRDA criteria for probable Alzheimer’s disease [34]. Exclusion criteria were CDR > 2, Hachinski Ischemia score >4 [35], MRI evidence of severe vascular brain lesions or evidence of other degenerative or secondary dementias.

The control group was composed of 13 subjects above 60 years of age, all recruited from the community-based study mentioned above [24, 25]. They underwent the same diagnostic evaluation described above including a structured interview, physical and neurological examination, laboratory workup and an MRI scan. Exclusion criteria were: diagnosis of cognitive impairment, no dementia (CIND) [36]; history of traumatic brain injury or stroke; dementia; and other neuropsychiatric disorders (aphasia, depression, dysthymia, mental retardation, anxiety disorders, alcohol dependence).

### Assessment of neuropsychiatric symptoms

Patients and their caregivers completed the NPI [7,37], covering 12 types of neuropsychiatric symptoms in dementia that might have been present in the last month. Each domain of symptoms is assessed using a 1–4 point scale to ascertain its frequency (occasionally, less than once a week, very frequently, or more than once a day) and a 1–3 point severity scale (mild, moderate, or severe intensity). Subsequently, each NPI domain score (range of 1–12) is reached by multiplying the frequency and severity scores, and an NPI total score is obtained by adding the scores from each domain.

AD subjects were dichotomized into two groups, those with few symptoms (2 or fewer) and those with multiple symptoms (3 or more).

### Magnetic resonance imaging data acquisition

Imaging data were acquired using a 1.5T GE Signa Horizon LX scanner (General Electric, Milwaukee, Wisconsin, USA).An axial gradient-echo T1-weighted images were obtained, with 1.5-mm thick sections, repetition time [TR, ms] = 21.7, echo time [TE, ms] = 5.2, flip angle = 20^o^, and matrix size 256x256x124).

### VBM: Image processing

Data were processed using Statistical Parametric Mapping software (SPM8) (Wellcome Department of Imaging Neuroscience, London), running in MATLAB R2010a (Mathworks, Sherborn, MA). In SPM8, an image from the control group with no significant atrophy was used as reference to realign images. The images were then segmented into gray matter (GM), white matter (WM), and cerebrospinal fluid (CSF) using the unified segmentation procedure in SPM8 [38]. The Diffeomorphic Anatomical Registration Through Exponentiated Lie Algebra (DARTEL) algorithm [39] was then used to spatially normalize the segmented images. These fully normalized images were resliced with trilinear interpolation to a final voxel size of 1.5×1.5×1.5 mm^3^. An additional “modulation” step was applied, consisting of multiplying each spatially normalized GM image by its relative volume before and after normalization; this ensured that the total amount of GM in each voxel was preserved [40]. We used the ‘normalize_DARTEL’ matlab script [41,42] to complete the affine transformation of segmented brain tissues into the MNI space. Finally, the resulting GM images were smoothed using an 8-mm isotropic kernel at full-width half-maximum to ensure a normal distribution of the data, as required by subsequent statistical parametric tests.

### VBM: Statistical analysis

The comparison between groups was carried out using two-sample t-tests in SPM8. A measure of the total intracranial volume (TIV) was entered as a potential confound in an analysis of covariance. The TIV was calculated from normalized modulated images through ‘get_totals.m’ function [43] available online (http://www.cs.ucl.ac.uk/staff/g.ridgway/vbm/) from the sum of the GM, WM and CSF segments throughout the whole brain [43]. In addition, voxel wise comparisons were masked using a comparison-specific explicit optimal threshold gray matter mask created using the Masking toolbox based on controls images [44]. Resulting statistics were thresholded at a p<0.001, uncorrected for multiple comparisons, with cluster size of 25 contiguous voxels and displayed as statistical parametric maps into standard Montreal Neurological Institute (MNI) space. Statistical inference was conducted in two steps. First, the familywise error (FWE)-corrected for multiple comparison over the whole brain was used, and findings were reported as significant at a stringent FWE-corrected p<0.05 statistical threshold. Second, the small volume correction (SVC) approach was used with the objective of restricting the total number of voxels included in the statistical analyses, allowing the inspection of each statistical map for the presence of suprathreshold clusters in brain regions where volume loss had been predicted *a priori*. Each region was defined by merging the right and left region-of-interest (ROI) masks from the Anatomical Automatic Labeling atlas included in the Pickatlas software [45,46,47]. ROI masks involved brain regions selected *a priori* due to their role in memory, emotional processing and salience brain networks as well as previous findings of AD-related neuroimaging abnormalities, including temporo-limbic areas, the lateral temporal cortex, parietal lobe, insula, frontal lobe and cingulate cortex [8,9,11,12,13,22]. Findings from the SVC-based analyses were considered significant if reaching *p*<0.05 peak level (FWE-corrected) over the respective ROI, with clusters including at least 25 voxels. In the whole-brain and SVC analyses, we converted MNI voxel coordinates of peak statistical significance to the Talairach and Tournoux (1988) system from the Talairach Daemon (TD) [48,49] as implemented in the PickAtlas software (http://fmri.wfubmc.edu/software/PickAtlas).

In addition to group comparisons, we also investigated the relationship between the NPI score (frequency *x* severity) for each individual neuropsychiatric symptom and regional gray matter volume MRI findings. The “multiple regression design” option on SPM8 was employed, using each individual NPI score as variable of interest and the measure of total intracranial volume (TIV) as nuisance variable. The same statistical inference approach described above was employed in such analyses.

### Demographic and clinical data: Statistical analysis

Kolmogorov-Smirnov a Test was used to verify the normal distribution of the variables. Chi-square Test (***χ***^**2**^) analyses the frequency of categorical variables. Relationships between cluster of symptoms and other independent variables were examined with t-test or non-parametric test (Mann-Whitney test), when variance homogeneity was not reached, using SPSS software package (version 16.0 for windows, SPSS Inc., Chicago, IL).

## Results

### Demographic and cognitive data

Table 1 shows demographic data and cognitive profiles of the AD and control groups. There were no significant between-group differences regarding sex or age, but AD patients displayed significantly lower mean years of education in comparison with healthy controls (3.42 ± 3.83 *versus* 6.23 ± 3.79). Expected significant differences were found between AD and controls in terms of MMSE, CAMCOG and CDR scores. Within the AD sample, there were no statistically significant differences between patients from the community-based study (n = 9; 47.4%) and AD patients recruited in the Old Age Psychiatry outpatients Dementia Clinic (n = 10; 52.6%).

**Table 1:** Demographic characteristics and cognitive profile of AD subjects, AD with few and multiple NPS, and healthy controls.

Groups						
	AD	AD with few NPS	AD with multiple NPS	*Statistical tests* *few vs multiple NPS*	Control	*Statistical tests* *AD vs Control*
N	19 (100%)	7 (36.8%)	12 (63.2%)		13	
Sex						
Female (%)	14 (73.7%)	6 (85.7%)	8 (66.7%)	*χ*^2^ = 0.827 p = 0.60	11 (84.6%)	*χ*^2^ = 0.54 p = 0.67
Age, years*						
Mean ± SD 95% CI	73.58 ± 7.791 (69.82–77.33)	72.71 ± 8.712 (64.66–80.77)	74.08 ± 7.561 (69.28–78.89)	(F = 0.404; p = 0.53) t test p = 0.72	69.54 ± 5.695 (66.10–72.98)	(F = 1.565; p = 0.22) t test p = 0.12
Min-Max	(61–86)	(62–85)	(61–86)		(62–80)	
Education, years						
Mean ± SD 95% CI	3.42 ± 3.834 (1.57–5.27)	1.43 ± 1,618 (-0.07–2.93)	4.58 ± 4,316 (1.84–7.33)	MW = 19.00; p = 0.05	6.23 ± 3.789 (3.94–8.52)	MW = 63.50; p = 0.02
MMSE*						
Mean ± SD 95% CI	17.21 ± 5.181 (14.71–19.71)	15.14 ± 4.488 (10.99–19.29)	18.42 ± 5.351 (15.02–21.82)	(F = 0.216; p = 0.65) t test p = 0.19	26.31 ± 3.301 (24.31–28.30)	(F = 1.776; p = 0.19) t test p≤0.001
CAMCOG						
Mean ± SD 95% CI	54.79 ± 6.851 (46.67–62.91)	51.71 ± 15.446 (37.43–66.00)	56.58 ± 18.028 (45.13–68.04)	MW = 28.500; p = 0.25	87.54 ± 11.362 (80.67–94.40)	MW = 4.00; p≤0.001
CDR						
0	0				13 (100.0%)	*χ*^2^ = 43,23 p≤0.001
0,5	4 (21.1%)	2 (28.6%)	2 (16.7%)		0	
1,0	7 (36.8%)	3 (42.9%)	4 (33.3%)		0	
2,0	8 (42.1%)	2 (28.6%)	6 (50%)	*χ*^2^ = 0.889 p = 0.64	0	

AD with few (≤2) and multiple (≥3) neuropsychiatric symptoms (NPS); SD = Standard deviation; CI = Confidence interval; MW = Mann-Whitney test; *χ*^2^ = Chi-square; MMSE = Mini Mental State Examination; CAMCOG = Cognitive Section of the CAMDEX; CDR = Clinical Dementia Rating Scale.

*Normal distribution on Kolmogorov-Smirnov test.

In addition, Table 1 shows demographic data and the cognitive profile of the two AD subgroups presenting few (2 or fewer symptoms; n = 7) or multiple (3 or more symptoms; n = 12) neuropsychiatric symptoms. There were no significant differences between the two subgroups regarding sex, age, years of education, MMSE and CAMCOG scores, or severity of dementia as measured by the CDR.

Twelve AD patients (63.2%) were evaluated with MRI before receiving any antidementia drugs or pharmacological treatment for neuropsychiatric manifestations. In the remaining AD sub-sample, 5 (26.3%) patients were treated with antidepressants, 2 (10.5%) with antipsychotics, 2 (10.5%) with benzodiazepines and 1 (5.3%) with an anticonvulsant. Three AD patients were using more than one psychotropic agent. Two (10.5%) AD patients were under use of anticholinesterase inhibitors, one of which concomitantly with treatment for neuropsychiatric symptoms. There were no differences between the AD subgroups with few *versus* multiple neuropsychiatric symptoms either in regard to the use of antidementia drugs (*χ*^2^ = 0.166; p = 1.00) or other psychotropic agents (*χ*^2^ = 0.83; p = 0.60).

### Neuropsychiatric symptoms

Table 2 shows the frequency of each neuropsychiatric symptom in the AD and control groups. There was a statistically significant difference between AD patients and controls in regard to the frequency of subjects presenting at least 1 neuropsychiatric symptom (100% in AD subjects, 38.5% in controls, *χ*^2^ = 15.590; p ≤ 0.001). In the AD group, the most frequent symptom was anxiety (78.9%), followed by apathy (68.4%), and irritability (52.6%). As expected, there were significant differences in NPI scores (Mann-Whitney test 0.00, p≤0.001) between the AD subgroup with few neuropsychiatric symptoms (mean = 5.714; SD 4.855; 95% CI 1.22–10.20; median 5.00) and the AD subgroup with multiple symptoms (mean = 46.50, SD 1.927; 95% CI 34.25–58.74; median 44.00).

**Table 2:** Percentage of subjects presenting with each neuropsychiatric symptom and mean NPI score in the AD and control groups.

Symptoms	AD Frequency Mean NPI score (SD)	Controls Frequency Mean NPI score (SD)
Anxiety	78.9% (15) 5.89 (4.42)	15.4% (2) 0.31 (0.85)
Apathy	68.4% (13) 5 (4.69)	7.7% (1) 0.46 (1.66)
Irritability	52.6% (10) 2.79 (3.91)	7.7% (1) 0.46 (1.66)
Sleep disorders	47.4% (9) 4.16 (4.89)	23.1% (3) 0.85 (1.91)
Disinhibition	42.1% (8) 2.05 (3.36)	0% (0)
Depression	36.8% (7) 1.89 (3.35)	15.4% (2) 0.54 (1.66)
Eating disorders	36.8% (7) 3.05 (4.90)	7.7% (1) 0.46 (1.66)
Agitation	31.6% (6) 2.05 (4.02)	15.4% (2) 0.23 (0.60)
Delusions	31.6% (6) 1.47 (2.46)	0% (0)
Aberrant motor behaviour	26.3% (5) 2.26 (4.27)	0% (0)
Hallucinations	21.1% (4) 0.58 (1.83)	0% (0)
Elation	5.3% (1) 0.21 (0.92)	0% (0)
Total	100% (19)	100% (13)

The distribution and combination of neuropsychiatric symptoms in the AD group are provided in S1 Table. Patients were divided into two subgroups according to the number of neuropsychiatric symptoms assigned on the NPI: subjects with few (≤2) (n = 7; 36.6%) or multiple (≥3) neuropsychiatric symptoms (n = 12; 63.4%).

There were no significant correlations between the NPI score of neuropsychiatric symptoms (NPI12) and cognitive test scores in the AD group, either when the MMSE (rho = 0.277, p = 0.252) or CAMCOG (rho = 0.236, p = 0,330) were used.

### Regional GM volume differences between AD patients and controls

**Fig 1:**
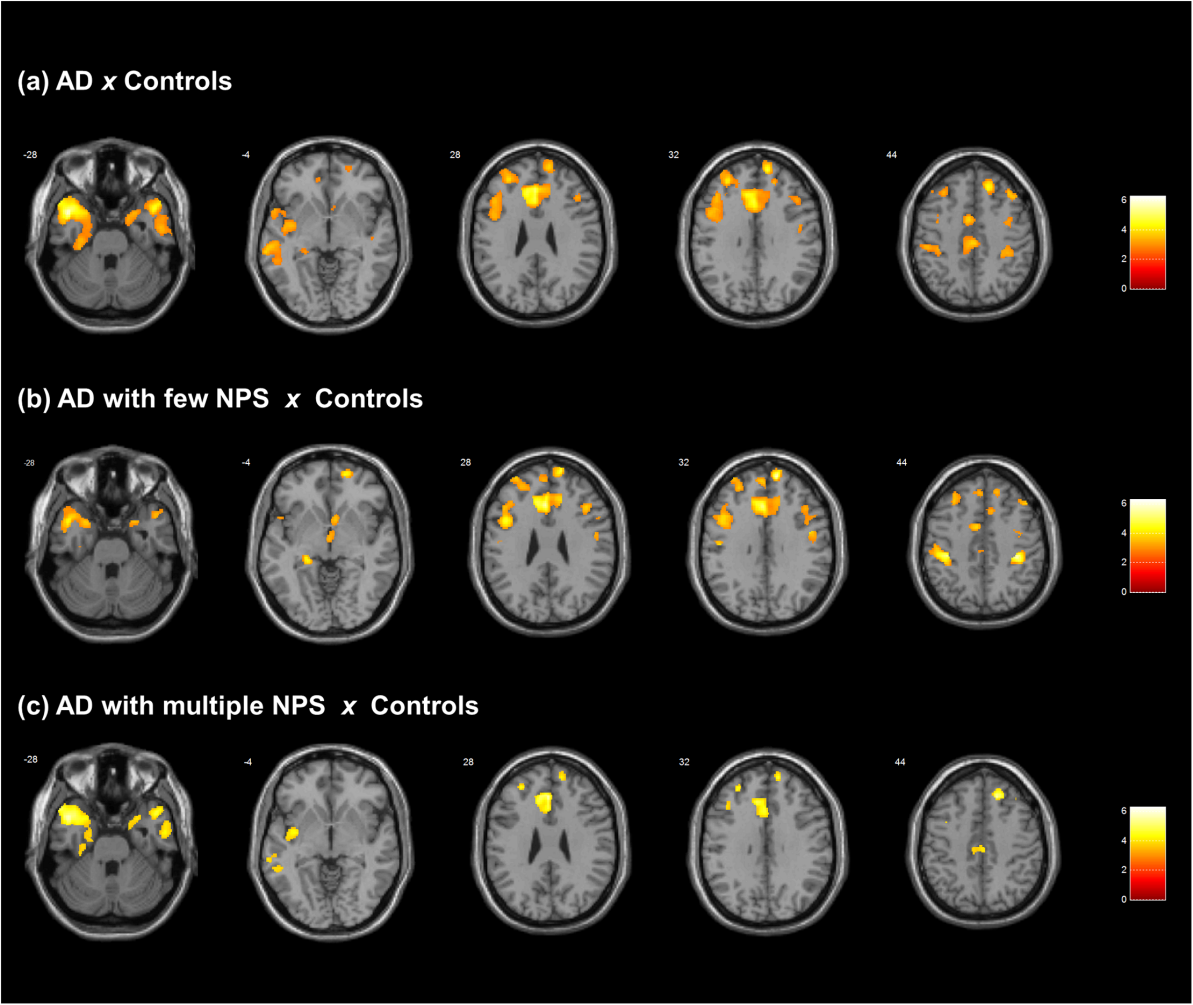
Findings of gray matter volume decreases in all AD patients (both groups) in comparison with healthy controls. Axial brain slices (T1 MNI template from SPM8) are overlaid by results from comparison between all AD patients, with few or with multiple neuropsychiatric symptoms, and healthy controls (with correction for multiple comparisons over the whole brain, p<0.05).The right hemisphere of the brain is shown on the right. Statistical parametric maps were thresholded at p<0.001 uncorrected for multiple comparisons, with a minimum cluster extent of 25 voxels. The figure was produced from visualized results using xjView toolbox (http://www.alivelearn.net/xjview). A) VBM analysis results showing GM reductions in the all AD patients (n = 19) *versus* controls (n = 13). Findings are highlighted in superior temporal gyrus extended to the middle temporal and parahippocampal gyri, anterior cingulate gyrus extended to the medial frontal gyrus, and superior-middle frontal gyri (significant at p < 0.05, FWE-corrected over the whole brain). B) VBM analysis results showing GM reductions in AD patients with few NPS (n = 7) *versus* controls (n = 13). Widespread clusters are shown involving the anterior cingulate cortex, prefrontal cortex and postcentral gyrus (significant at p < 0.05, FWE-corrected over the whole brain) (Table 3). Less robust findings are shown in the left temporal cortex and temporo-limbic region (significant in SVC analyses at p<0.05 FWE-corrected; Table 4). C) VBM analysis results showing GM reductions in AD patients with multiple NPS (n = 12) *versus* controls (n = 13). Findings are highlighted in the left insula and temporal cortex (significant at p < 0.05, FWE-corrected over the whole brain) (Table 3). Additional clusters are seen in the anterior cingulate and prefrontal cortices (significant only in SVC-based analyses at p<0.05 FWE-corrected) (Table 5), and these differences appeared to be less widespread relative to the findings detected in the AD patients with few NPS.

Fig 1 demonstrates those brain regions that showed significantly lower regional GM volumes in AD patients in comparison to healthy controls in the exploratory whole-brain analysis (p<0.05, corrected for multiple comparisons over the whole brain). There were clusters in the following regions: 1) superior temporal gyrus that extended to the middle temporal and parahippocampal gyri (Left hemisphere: BA38, MNI coordinates *x y z* -43 10–31, k = 8480, Z score = 5.10, p<0.001 FWE-corrected peak; Right hemisphere: MNI coordinates *x y z* 47 14–30, k = 3681, Z score = 4.71, p = 0.023 FWE-corrected peak); 2) anterior cingulate gyrus that extended to the medial frontal gyrus (Left hemisphere: BA32, MNI coordinates *x y z* -8 33 26, k = 2679, Z score = 4.92, p = 0.020 FWE-corrected peak); and 3) superior and middle frontal gyri (Left hemisphere: BA6, MNI coordinates *x y z* -31 27 37, k = 2943, Z score = 4.85, p = 0.008 FWE-corrected peak; Right hemisphere: BA10, MNI coordinates *x y z* 13 55 34, k = 1243, Z score = 4.69, p = 0.011 FWE-corrected peak).

### Differences in regional GM volumes between AD subjects with few or multiple neuropsychiatric symptoms relative to healthy controls

Table 3 displays the significant results of exploratory whole-brain analyses (p≤0.05, corrected for multiple comparison over the whole brain) comparing AD patients with few or multiple neuropsychiatric symptoms to healthy controls. Relative to controls, the AD subgroup with few neuropsychiatric symptoms displayed four clusters of significantly decreased GM volume: the right superior frontal gyrus (extended to medial frontal gyrus), the left anterior cingulate gyrus (extended to medial frontal gyrus) and the postcentral gyrus bilaterally (extended to inferior parietal lobule and precentral gyrus). In the AD subgroup with multiple neuropsychiatric symptoms, there was one cluster of significantly reduced GM volume relative to controls in the left superior temporal gyrus (extended to parahippocampal gyrus, middle temporal gyrus, and insula). In summary, the AD subgroup with few neuropsychiatric symptoms displayed comparatively more extensive GM decreases in the anterior cingulate gyrus, frontal lobe, and parietal lobe relative to controls (see Fig 1). The AD subgroup with multiple neuropsychiatric symptoms showed more extensive GM decreases involving the superior temporal gyrus and insula relative to controls (see Fig 1).

**Table 3:** Significant findings of gray matter volume decreases in AD patients with few or multiple neuropsychiatric symptoms in comparison with healthy controls (with correction for multiple comparisons over the whole brain, p<0.05).

Brain region (BA)^a^	Hemisphere	MNI coordinates^b^ *x y z*	K^c^	Z score^d^	p (FWE corr) ^e^ Peak-level
*AD with few NPS (n = 7) versus controls (n = 13)*					
Superior frontal gyrus (BA9) (extended to medial frontal gyrus)	Right	13 55 34	443	5.10	0.009
Anterior cingulate gyrus (BA32) (extended to medial frontal gyrus)	Left	-2 25 28	2173	4.92	0.019
Postcentral gyrus (BA2) (extended to inferior parietal lobule)	Right	37–30 44	437	4.85	0.024
Postcentral gyrus (BA2) (extended to inferior parietal lobule and precentral gyrus)	Left	-38–28 46	807	4.69	0.044
*AD with multiple NPS (n = 12) versus controls (n = 13)*					
Superior temporal gyrus (BA38) (extended to parahippocampal gyrus, middle temporal gyrus, and insula)	Left	-44 10–31	3709	4.71	0.032

a BA = Approximate Brodmann Area.

b Voxel coordinates of maximal statistical significance in the cluster.

c Total voxels number in each cluster with Z>3.09 (corresponding to P≤ 0.001) and k>25

d Z scores for the voxel of maximal statistical significance in each cluster.

e Statistical significance after correction for multiple comparisons at voxel level

Additional foci of between-group differences emerged when the more flexible SVC-based (small volume correction) statistical approach of adjustment for multiple comparisons was applied to the brain regions predicted *a priori* to show significant findings. The emergence of these additional foci of significant GM volume deficits led to a greater degree of similarity between the two AD subgroups as compared to controls (see Tables 4 and 5). Thus, in the AD subgroup with few neuropsychiatric symptoms relative to controls, the SVC-based analyses revealed additional findings of significantly reduced GM volumes in the parahippocampal gyrus, superior and middle temporal gyri (Table 4). Conversely, in the AD subgroup with multiple neuropsychiatric symptoms relative to controls the SVC-based analyses showed additional foci of significantly reduced GM volumes in the anterior cingulate gyrus, medial and middle frontal gyri (Table 5).

**Table 4:** Additional findings of gray matter volume decreases in AD patients with few neuropsychiatric symptoms in comparison with healthy controls, corrected for multiple comparisons using the flexible, small-volume correction (SVC) method.

Brain region (BA)^a^	Hemisphere	MNI coordinates^b^ *x y z*	K^c^	Z score^d^	p (FWE corr) ^e^ Peak-level
Parahippocampal gyrus—Uncus (BA38)	Right	24 9–32	167	3.81	0.021
Superior temporal gyrus (BA38)	Left	-43 10–31	545	4.26	0.004
	Right	47 15–28	106	3.57	0.038
Middle temporal gyrus (BA21)	Left	-44 7–29	105	4.04	0.031
	Left	-66–19–9	248	4.00	0.035

a BA = Approximate Brodmann Area.

b Voxel coordinates of maximal statistical significance in the cluster.

c Total voxels number in each cluster with Z>3.09 (corresponding to P≤ 0.001) and k>25

d Z-score for the voxel of maximal statistical significance in each region.

e Statistical significance after correction for multiple comparisons at the voxel level

**Table 5:** Additional findings of gray matter volume decreases in AD patients with multiple neuropsychiatric symptoms in comparison with healthy controls, corrected for multiple comparisons using the flexible, small-volume correction (SVC) method.

Brain region (BA)^a^	Hemisphere	MNI coordinates^b^ *x y z*	K^c^	Z score^d^	p (FWE corr)^e^ Peak-level
Anterior cingulate gyrus (BA32)	Left	-8 35 24	500	4.22	0.004
Medial frontal gyrus (BA9)	Right	16 34 45	209	4.28	0.015
Middle frontal gyrus (BA9)	Left	-33 27 39	154	4.36	0.009
	Left	-38 4 52	122	4.04	0.028

a BA = Approximate Brodmann Area.

b Voxel coordinates of maximal statistical significance in the cluster.

c Total voxels number in each cluster with Z>3.09 (corresponding to P≤ 0.001) and k>25

d Z-score for the voxel of maximal statistical significance in each region.

e Statistical significance after correction for multiple comparisons at the voxel level

### Relationship between individual neuropsychiatric symptoms and regional GM volumes

Exploratory whole-brain analyses showed no significant relationship between the presence of each neuropsychiatric symptom and GM volumes in the AD group (p ≤ 0.05, corrected for multiple comparisons over the whole brain). The only neuropsychiatric manifestation that could not be evaluated in such analyses was elation, as this symptom was present in only one individual.

When these analyses were repeated using the SVC approach, the presence of sleep disorders was correlated to decreased GM volume in a cluster involving the right middle temporal pole (BA 38) extending towards the middle and inferior temporal gyri (MNI coordinates *x y z* 37 20–41, k = 269, Z score = 3.84, p<0.05 FWE-corrected peak).

## Discussion

This VBM study identified the presence of decreased GM volume in association with neuropsychiatric symptoms in AD sufferers. While manifestations of psychosis, agitation, hyperactivity, disinhibition and aberrant motor behaviour were present in combination in the majority of AD individuals from the subgroup of AD patients with high NPI scores, the other AD subgroup had few neuropsychiatric symptoms, predominantly apathy, irritability or anxiety. Our results indicated the presence of different patterns of lower GM volume in those two AD subgroups relative to healthy controls.

Previous clinical studies of AD have suggested the possible relevance of grouping neuropsychiatric manifestations into separate syndromes rather than investigating individual symptoms [21,50,51]. Variable statistical techniques have been employed in such studies to explore patterns of correlation between variables and to group them into factors or classes characterizing the profile of neuropsychiatric syndromes in dementia [16,19,20,52,53,16,54,55]. A recent review indicated that neuropsychiatric symptoms have often been grouped into clusters of affective, psychotic, hyperactivity and euphoria features [17]. However, there have been variations across studies in regard to the composition of syndromes, with symptoms such as anxiety, agitation and even delusions being present in more than one syndrome in some investigations [17,19]. Moreover, a considerable degree of co-occurrence of more than one syndrome in the same AD patient has been reported [20]. Such literature findings indicate that alternative models, such as the one employed herein, may be fruitful in neurobiological investigations of the neural substrates of neuropsychiatric symptoms associated with AD.

The patterns of decreased GM volume identified in our AD subgroup with multiple neuropsychiatric symptoms relative to controls indicate that although different neuropsychiatric manifestations may have distinct psychopathological characteristics (and possibly etiological influences), these symptoms may co-occur in the same AD patients and share the same underlying neurodegenerative GM changes. Support to this proposition comes not only from real-world clinical practice but also from studies that have shown that in regard to neuropsychiatric manifestations, patients with dementia may be categorized into those with complex disorders with multiple symptoms versus those minimally symptomatic or with few affective symptoms [20,21]. Also, other studies have shown a high degree of inter-correlation between separate behavioral syndromes in samples of patients with dementia [51]. Previous structural neuroimaging studies have also shown superimposing results regarding associations between dementia-related neuropsychiatric symptoms and brain atrophy patterns [8,9,12,13,56], with the same brain areas being implicated for instance in association with both apathy and agitation, or with both agitation and psychosis [57]. Finally, the paucity of significant findings in the VBM analyses carried out for each neuropsychiatric symptom individually in the current study also give credence to the approach of grouping AD individuals according to the presence of multiple versus few neuropsychiatric symptoms.

As demonstrated in the VBM analysis using the strict statistical threshold corrected for multiple comparisons over the whole brain, AD patients with multiple neuropsychiatric manifestations presented more evident GM volume differences in the insula and left superior temporal gyrus. The insula is a key component of the salience network, which has been proposed to play a central role in the detection of potentially relevant stimuli and the triggering of appropriate behavioural responses of an individual, processes that demand the integration of several brain systems (sensory, attentional, visceral, and autonomous) [58]. There is evidence that the abnormal functioning of the salience network may contribute to deficits in processes of attentional, affective and social control in other neuropsychiatric disorders [58]. For example, reduced within-network connectivity and failure of salience network hubs in communicating with additional brain networks such as the default-mode and executive-control networks, via insular impairments, are thought to be critical to the pathophysiology of schizophrenia and bipolar affective disorder [59,60]. The insular cortex, particularly implicated in the above cited neuropsychiatric disorders, is the first cortical target of viscerosensorial and interoceptive inputs, and it is considered critical to the generation of subjective feelings that orient decision-making [58]. The superior temporal gyrus is another area commonly implicated in the emergence of complex psychopathological manifestations. Structural and functional abnormalities of the superior temporal gyrus have been related, for instance, to schizophrenia as a whole and specifically with psychotic symptoms such as hallucinations [61]. This brain region has also been implicated in prodromal cases of schizophrenia [62]. Finally, it is relevant to note that the GM volume deficits most prominent in the AD patients with multiple neuropsychiatric manifestations were lateralized to the left hemisphere (both in the insula and superior temporal gyrus). Such pattern of morphological lateralization has also been reported in the brains of individuals with schizophrenia [63]. In summary, AD patients with multiple neuropsychiatric symptoms showed associations with brain regions which regulate the complex integration of the multiple brain systems linked to human behavioral responses. These behavioral responses could be psychopathological complexes where as they enroll the manifestation of multiple symptoms at same time such as psychoses, hyperactivity and agitation syndromes.

In contrast with the above findings in multiple-symptom subjects, the VBM whole brain analyses showed that AD subjects with few neuropsychiatric symptoms had more extensive GM volume differences in prefrontal regions that comprise the central executive network, as well as in the dorsal anterior cingulate and post-central gyri. The anterior cingulate cortex coordinates executive and auto-monitoring systems through coactivation of specific sensory networks and associative networks, such as the dorsolateral prefrontal and posterior parietal attentional networks, with connections to subcortical nuclei including the striatum. The cortico-subcortical circuit connecting the anterior cingulate gyrus and the striatum is responsible for integrating emotionally relevant tone with executive functioning [64]. As described in detail previously by Menon et al. [65], the central executive network corresponds to a frontoparietal system anchored in the dorsolateral prefrontal and posteriolateral parietal cortices, and this network is thought to be crucial for actively maintaining and manipulating information in working memory, for rule-based problem solving, and for decision making in the context of goal-directed behavior. Abnormalities in the central executive network may contribute to symptoms such as apathy [66]. Impaired functioning of fronto-subcortical circuits has been proposed to elicit behavioural abnormalities in AD including agitation and apathy through deficits in anticipatory aspects of anxiety and problem-solving [57]. Finally, the postcentral gyrus corresponds to the somatosensory cortex, which is involved in subjective experience from emotion perception and interoceptive process [67]. The understanding of interoception has been recently changed from a more restricted view solely related to the mapping of visceral sensations to an expanded concept related to phenomenological experiences of bodily states produced within the central nervous system regardless of the source from which the information used to build such experience emerges [68]. It has been suggested that in the presence of ambiguous sensory stimuli and noise that reduce perceptual accuracy in homeostatic pathways, the brain increases top-down modulation and creates a biased self-referential perception of what is occurring within the body [69]. Information regarding interoceptive and subjective emotional states which are processed inaccurately could contribute to maladaptative responses such as heightened reactivity to threat uncertainty [70] resulting in anxiety or irritability reactions. Consequently, the above-mentioned brain areas could be associated not only with presence of few neuropsychiatric symptoms, but mainly with reactional psychopathologic features from anxiety spectrum. Therefore, AD patients with few neuropsychiatric symptoms showed loss gray matter in the areas of the central executive network and the somatosensory cortex because those areas are involved in the interoception regulation and problem-solving process contributing for reactional behavioral changes as anxiety.

One important aspect of our findings is that the presence of multiple neuropsychiatric manifestations was not associated with more significant or more widespread GM volume deficits in AD. To our knowledge, no previous MRI study carried out the same comparison between AD patients with few or multiple neuropsychiatric symptoms against controls, as reported herein. Our findings suggest that the presence of multiple neuropsychiatric symptoms is more related on the degree of atrophy in specific brain networks rather than dependent on the global severity of widespread neurodegenerative brain changes.

Despite the separate associations of few and multiple neuropsychiatric symptoms with distinct topographical patterns of lower GM volume as discussed above, it should be noted that more similarities in the topography of GM volume decrements across the two AD subgroups emerged when the more flexible SVC (small-volume corrected) statistical threshold was used (see Tables 4 and 5). This may indicate that the distinction of GM volume deficits in specific brain regions between AD patients with few or multiple neuropsychiatric symptoms was one of degree rather than kind. Also, it should be noted that key brain regions highlighted herein, namely the insula and anterior cingulate cortex (BA32), have overlapping roles in the mental processes discussed above. Thus, the anterior cingulate cortex (which displayed more prominent GM deficits in the AD subgroup with few neuropsychiatric symptoms) is as relevant to the salience network as the insula, the involvement of which was discussed above as possibly critical to the emergence of multiple behavioural manifestations in the subgroup with multiple neuropsychiatric symptoms. Conversely, the higher-order representation of body states (discussed in the context of anxiety and irritability as manifestations seen in AD patients with few neuropsychiatric manifestations) is thought to demand inputs from the posterior insula as well as the somatosensory, visual, auditory and vestibular cortices, which are integrated in the middle insular cortex [71].

Mechanisms to explain different topographical trajectories of brain changes associated with the presence of neuropsychiatric manifestations in AD subjects are not readily attainable. Since the presence of multiple neuropsychiatric manifestations was neither related to more widespread brain volume deficits nor to more severe cognitive deficits in our study, it is unlikely that the presence of such neuropsychiatric symptoms could be explained by more severe or widespread brain degenerative changes. One other possibility is that region-specific atrophy related to neuropsychiatric symptoms could be consequential to neurochemical deficits [72]. For instance, changes in dopaminergic pathways, usually implicated in the emergence of psychotic symptoms, have been associated with changes in brain morphology [73,74]; such changes may affect selected brain regions such as the insula and superior temporal gyrus implicated in the current study, among others [74]. One recent neuropathological investigation reported correlations between the severity of neuropsychiatric symptoms in AD patients and postmortem levels of different monoamines and their metabolites in the hippocampus, thalamus, superior temporal gyrus and cerebellum, although there were variable results depending on the type of neuropsychiatric manifestation rated [75]. Finally, region-specific brain volume deficits associated with neuropsychiatric symptoms could be related to distinct patterns of protein pathology in AD. Postmortem investigations have suggested that the presence of psychotic symptoms, for instance, may be more directly related to tau rather than beta-amyloid pathology [72]. Distinct neuronal pathways and their connections may be differentially affected by the deposition of hyperphosphorilated tau or beta-amyloid peptide [76,77], and such distinctions may influence on the emergence of neuropsychiatric manifestations [76,78]. Deposition of hyperphosphorilated tau in limbic and frontal cortical neurons seems to be associated with neuropsychiatric symptoms including agitation, aberrant motor behavior, apathy, and psychosis [79,80,81]. In future investigations, it will be important to investigate associations of multiple neuropsychiatric symptoms concurrently with regional brain imaging findings and cerebrospinal fluid markers of tau and beta-amyloid pathology.

One of our study strengths is the use of an alternative approach for investigating neuropsychiatry symptoms in AD which is nearer to real-world clinical manifestation than applying computational analysis techniques where the syndromes co-occurrence is not considered. Our results reinforce VBM’s contribution to research on the neurobiological underpinnings of neuropsychiatric symptoms in AD. Limitations of this study include the modest sample size; replication of our preliminary results is therefore warranted using larger samples.

In conclusion, the results of this VBM study indicate differences between AD patients who have few versus multiple neuropsychiatric symptoms in regards to the degree of atrophy in specific brain areas. Our findings suggest that the presence of multiple neuropsychiatric symptoms is more related to the degree of atrophy in specific brain networks rather than dependent on the global severity of widespread neurodegenerative brain changes. These findings help to expand the understanding about neurobiological aspects of AD neuropsychiatric symptoms. Further neuroimaging studies are needed to clarify which mechanisms are involved in the complex neuropsychiatric manifestations observed in dementia patients.

## Supporting information

S1 Table Distribution and combination of neuropsychiatric symptoms in the Alzheimer´s disease (AD) group. (DOCX)

S2 Table Demographic and clinical data from subjects. AD = Alzheimer´s Disease; MMSE = Mini Mental State Examination; CAMCOG = Cognitive Section of the CAMDEX; CDR = Clinical Dementia Rating Scale; NPI = Neuropsychiatric Inventory; Number_of_nps = number of neuropsychiatric symptoms that were present in the subject. *For each symptom presence variable, 1 means presence of the referred symptom, and 0 means absence of the symptom. ** Symptoms scores of each domain were calculated by multiplying their frequency and severity scores.
(XLSX)
