# Supplementary material for: Cortical brain volume abnormalities associated with few or multiple neuropsychiatric symptoms in Alzheimer’s disease
Source: PLoS One. 2017 May 8;12(5):e0177169. doi: 10.1371/journal.pone.0177169 (PMC5422036; doi:10.1371/journal.pone.0177169)
Supplement: S1 Table — (DOCX) [file pone.0177169.s001.docx]

**S1 Table.** Distribution and combination of neuropsychiatric symptoms in the Alzheimer´s disease group

| **Number of NPS** | **Number of patients** | **%** | **Subject ID** | **Distribution and combination of NPS** |
| --- | --- | --- | --- | --- |
| ***1*** | 5 | 26.3 |  |  |
|  |  |  | 1 | Anxiety |
|  |  |  | 2 | Apathy |
|  |  |  | 3 | Anxiety |
|  |  |  | 4 | Apathy |
|  |  |  | 5 | Sleep disorders |
| ***2*** | 2 | 10.5 |  |  |
|  |  |  | 6 | Anxiety, and Eating disorders |
|  |  |  | 7 | Apathy, and Irritability |
| ***3*** | 0 | 0 |  |  |
|  |  |  | none |  |
| ***4*** | 1 | 5.3 |  |  |
|  |  |  | 8 | Hallucinations, Anxiety, Apathy, and Irritability |
| ***5*** | 1 | 5.3 |  |  |
|  |  |  | 9 | Hallucinations, Anxiety, Apathy, Irritability, and Sleep disorders |
| ***6*** | 3 | 15.8 |  |  |
|  |  |  | 10 | Delusions, Depression, Anxiety, Irritability, Sleep disorders, and Eating disorders |
|  |  |  | 11 | Depression, Anxiety, Apathy, Disinhibition, Irritability, and Eating disorders |
|  |  |  | 12 | Delusions, Depression, Anxiety, Apathy, Disinhibition, and Aberrant motor behaviour |
| ***7*** | 4 | 21.1 |  |  |
|  |  |  | 13 | Delusions, Depression, Anxiety, Elation, Apathy, Disinhibition, and Sleep disorders |
|  |  |  | 14 | Hallucinations, Agitation, Depression, Anxiety, Apathy, Sleep disorders, and Eating disorders |
|  |  |  | 15 | Hallucinations, Agitation, Anxiety, Disinhibition, Irritability, Aberrant motor behavior, and Sleep disorders |
|  |  |  | 16 | Agitation, Depression, Anxiety, Apathy, Disinhibition, Irritability, and Aberrant motor behavior |
| ***8*** | 1 | 5.3 |  |  |
|  |  |  | 17 | Delusions, Agitation, Anxiety, Apathy, Disinhibition, Irritability, Sleep disorders, and Eating disorders |
| ***9*** | 1 | 5.3 |  |  |
|  |  |  | 18 | Delusions, Agitation, Anxiety, Apathy, Disinhibition, Irritability, Aberrant motor behavior, Sleep disorders, and Eating disorders |
| ***10*** | 1 | 5.3 |  |  |
|  |  |  | 19 | Delusions, Agitation, Depression, Anxiety, Apathy, Disinhibition, Irritability, Aberrant motor behavior, Sleep disorders, and Eating disorders |
| **Total** | **19** | **100%** |  |  |
